# Symptoms and Quality of Life: Before and after stem cell transplantation in cancer

**DOI:** 10.12669/pjms.293.3290

**Published:** 2013

**Authors:** Ozlem Ovayolu, Nimet Ovayolu, Emine Kaplan, Mustafa Pehlivan, Gulendam Karadag

**Affiliations:** 1 **Ozlem Ovayolu, RN, PhD, Gaziantep University, Faculty of Health Science, Gaziantep, Turkey.**; 2 **Nimet Ovayolu, RN, PhD, Associate Professor, Gaziantep University, Faculty of Health Science, Gaziantep, Turkey.**; 3 **Emine Kaplan, Research Assistant, Adiyaman University, School of Health, Adiyaman, Turkey.**; 4 **Mustafa Pehlivan, MD, Associate Professor, Department of Hematology, Faculty of Medicine, Gaziantep University, Gaziantep, Turkey.**; 5 **Gulendam Karadag, RN, PhD,Gaziantep University, Faculty of Health Science, Gaziantep, Turkey.**

**Keywords:** Nursing, Stem Cell Transplantation, Symptoms, Turkey, Quality of Life

## Abstract

***Objective:*** This study was conducted thinking that it was extremely important in terms of the disease and treatment to assess the symptoms that may be encountered before and after a stem cell transplantation and quality of life.

***Methodology:*** A prospective longitudinal design was used.The study was completed in two years on 82 patients who underwent transplantation at the bone marrow transplantation unit. Data were collected using a questionnaire, the Edmonton Symptom Assessment Scale, and the Short Form-36 quality of life scale.

***Results:*** It was observed that the patients had low mean scores of physical and mental quality of life both before and after transplantation; there was an increase in the mean scores of all the symptoms and primarily of fatigue after the stem cell transplantation as compared to before it; and the mean scores of physical and mental quality of life further declined (p<0.05).

***Conclusion:*** Quality of life of patients who underwent stem cell transplantation is adversely affected in the periods immediately before and after transplantation. Patients’ quality of life worsens as the severity of symptoms experienced by patients increases.

## INTRODUCTION

Quality of life measures are subjective, reflecting the individual’s assessment of his/her life at one time relative to his/her previous state and prior experiences. After all, it is the patient’s own evaluation of his or her quality of life that is important. The patient’s perception of his own quality of life may be especially poignant after BMT. Many of the patients are happy or satisfied simply with the fact that they are alive following such intensive therapy. It may be that a patient’s perception of what is important for their global quality of life is simply life itself, the good and the bad. Community reintegration problems, which included difficulty in returning to former social roles, separation from home, family, and friends, difficulty in resuming social relations, dealing with stigmatization, problems with family and children, and financial and employment difficulties.^1^

Besides these problems, patients are often faced with other pre- or post-transplantation physical problems such as neutropenia, infection, bleeding, fatigue, nausea-vomiting, dehydration, diarrhea and mucositis.^2^ Thus, patients should be assessed by nurses and other members of health staff in physical, mental and social terms to identify possible problems and to support their quality of life. When making this assessment, it is very important for nurses to use their diagnosing skills, to discover unexpected/sudden reactions in patients on time^3^ and to administer appropriate nursing interventions without delay. Such interventions will not only control symptoms in a timely manner, but will also have positive effect on patients’ quality of life. This study was performed to identify pre- and post-transplantation symptoms in patients who underwent stem cell transplantation and to assess their quality of life.

## METHODOLOGY

***Design and Sample: ***A prospective longitudinal design was used. The study was completed in two years on 82 patients who underwent transplantation at the bone marrow transplantation unit of an oncology hospital located in the Southeastern Anatolia Region of Turkey. Eligibility criteria included being 17 years of age or older, speaking Turkish language, not having any known psychiatric disorders that would interfere with completion of the scales, not suffering from auditory or visual impairment, being able to answer all the questions, having a single transplantation, and being willing to participate in the study. Sixty three patients who did not meet the study criteria (those who refused to take part, were not able to communicate, had a poor general condition, were lost to follow-up, or developed a graft versus host disease) were excluded from the study.

***Data collection: ***The study data were collected using a questionnaire, the Edmonton Symptom Assessment Scale (ESAS), and the Short Form (SF)-36 quality of life scale. The questionnaire and the scales were administered a month prior to transplantation when the side effects of treatments were experienced intensely; ESAS and SF-36 quality of life scale were administered once more to the patients a month after transplantation.

***Questionnaire: ***The questionnaire included questions about some characteristics of the patients and their disease and treatment-related characteristics. Information on body weight and height were obtained by patients’ self-report. Body Mass Index (BMI) was calculated as weight (kilograms) divided by square of height (meters) and values of 18.5 and lower were classified as “underweight”, 18.5 to <25 as “normal weight”, >25 as “overweight”, and >30 as “obese”.^4^

***Edmonton Symptom Assessment Scale: ***The scale was developed by Bruera and associates^5^ and its validity and reliability for Turkish people were tested by Sadirli and Unsar.^6^ This tool is designed to assist in the assessment of nine symptoms commonly found in cancer patients: pain, fatigue, nausea, depression, anxiety, drowsiness, loss of appetite, decreased sense of well-being and shortness of breath. The ESAS also includes a section labeled “Other Problems”, to which three more symptoms that were detected by Sadirli and Unsar and listed as additional symptoms by patients were added as skin and nail changes, stomatitis, and numbness in the hands. The severity of each symptom at the time of assessment was rated from 0 to10 on a numerical scale, with “0” meaning that the symptom was absent, and “10” meaning that the symptom was of the worst possible severity.^7^

***Short Form-36 quality of life scale: ***The SF-36 was developed by Ware & Sherbourne^8^ as a comprehensive measure of general health status for use in the Medical Outcomes Study. The Turkish version of SF-36 is composed of 36 items; it measures eight dimensions of HRQoL and yields scores for each of these. The scale’s score may vary from 0-worst possible health status or quality of life to 100-best possible health status or quality of life. The SF-36 survey yields two comprehensive HRQoL indexes: the PCS (the first four domains) and the MCS (the last four domains).^9^

***Ethical considerations: ***Consent was obtained from the patients who were included in the study after they were provided with necessary explanation about the study’s objectives. Permission was received from the institution where the research was conducted and approval was obtained from the Ethical Committee of Faculty of Medicine, Gaziantep University.

***Data analysis: ***Descriptive statistics were reported as frequencies, means and standard deviations. Student t-tests, ANOVA, Mann Whitney U and Kruskal-Wallis tests were used to evaluate the relationship between some characteristics and disease and treatment statuses of the patients and their mean scores of physical and mental quality of life prior to stem cell transplantation. Additionally, Paired Sample t-test was used to compare physical and mental mean scores of ESAS and SF-36 quality of life scale before and after the stem cell transplantation and correlation analysis to evaluate the relationship between the symptoms and quality of life score. Statistically significant levels were set at p<0.05.

## RESULTS

The patients had low mean scores of both physical and mental quality of life. Those in 57 and over age group, women, those who were illiterate, those married, those who had economic difficulties and those who were obese had lower mean scores of physical and mental quality of life (Table-I).

**Table-I:** Comparison of specific characteristics of patients with their mean scores of physical and mental quality of life before stem cell transplantation

*Parameters*	*Quality of Life*		
		*PCS*	*MCS*
	*n(%)*	*Mean±SD*	*Mean±SD*
Age groups			
17-26 27-36 37-46 47-56 57 or over	26(31.7) 18(22.0) 13(15.9) 15(18.3) 10(12.2)	58.8±19.8 48.6±14.6 38.8±13.9 46.8±18.5 33.4±13.4 p=0.002	47.1±17.0 36.8±11.2 29.5±8.1 37.1±16.9 28.2±10.2 p=0.002
Gender			
Female Male	26(31.7) 56(68.3)	42.4±20.6 50.7±17.4 p=0.050	35.6±18.0 39.0±14.1 p=0.111
Education level			
Illiterate Primary school High school University	14(17.1) 36(43.9) 24(29.2) 8(9.8)	33.7±12.2 44.8±16.4 58.4±19.2 57.7±16.9 P=0.000	28.6±8.7 34.2±12.5 45.6±16.7 47.6 ±18.6 p=0.002
Marital status			
Married Single	60(73.2) 22(26.8)	43.8±17.2 59.7±18.0 P=0.001	35.0±14.1 45.9±16.1 p=0.002
Economic difficulties			
Yes No	47(57.3) 35(42.7)	44.8±18.2 52.6±18.7 P=0.062	34.9±13.7 41.9±16.8 p=0.041
Occupation			
Civil servant Worker Self-employed Unemployed Other	7(8.5) 9(11.0) 25(30.5) 13(15.9) 28(34.1)	48.1±14.5 48.4±12.4 47.3±16.5 63.9±20.5 41.4±19.1 p=0.075	39.3±15.6 35.1±8.4 36.0±13.1 50.9±18.3 34.2±15.3 p=0.092
BMI			
Underweight Normal Overweight Obese	3(3.7) 53(64.6) 19(23.2) 7(8.5)	52.8±33.3 49.7±17.9 46.8±19.5 37.7±17.3 p=0.452	41.0±26.7 38.3±14.9 38.0±15.3 33.3±17.7 p=0.720
Total	82(100.0)	48.1±18.7	37.9±15.4

n: number

MCS: Mental component summary, PCS: physical component summary.

SD: Standard deviation.

**Table-II:** Comparison of specific disease- and treatment related characteristics of patients with their mean scores of physical and mental quality of life before stem cell transplantation

*Parameters*	*Quality of Life*		
		*PCS*	*MCS *
	*n(%)*	*Mean±SD*	*Mean±SD*
Diagnosis			
Acute lymphocytic leukemia Acute myeloid leukemia Hodgkin lymphoma Non Hodgkin lymphoma Multiple myeloma Aplastic anemia	10(12.2) 26(31.7) 6(7.3) 13(15.8) 14(17.1) 13(15.9)	46.2±23.0 48.1±17.1 41.5±20.8 48.9±15.0 43.7±21.1 56.7±18.6 p=0.420	36.8±18.7 38.0±13.9 35.4±17.0 34.9±13.0 38.6±18.7 42.2±15.2 p=0.650
Time of diagnosis			
Less than 6 months More than 6 months	17(20.7) 65(79.3)	51.7±19.1 47.2±18.7 p=0.369	39.5±15.3 37.5±15.5 p=0.400
Stage			
I II III IV	11(13.4) 21(25.6) 29(35.4) 21(25.6)	45.8±22.6 49.2±19.1 47.7±16.8 48.7±20.0 p=0.944	37.0±17.4 38.1±15.7 36.4±13.9 40.3±16.8 p=0.839
Type of transplantation			
Autologous transplantation Allotransplantation	35(42.7) 47(57.3)	40.3±16.9 53.9±18.1 p=0.001	33.1±15.0 41.5±14.8 p=0.014
Opinion on the disease			
Easily treatable Requires long-term treatment	21(25.6) 61(74.4)	49.5±19.1 47.6±18.8 p=0.815	40.0±16.0 37.2±15.2 p=0.510
Adaptation to the disease			
Yes No	82(100.0) -	48.1±18.7 -	37.9±15.4 -
Change in body image			
Yes No	11(14.4) 71(86.6)	47.4±12.9 48.2±19.5 p=0.902	35.3±7.8 38.3±16.2 p=0.576
Worries about the future			
Yes No	53(64.6) 29(35.4)	45.5±19.0 52.9±17.6 p=0.105	36.0±14.9 41.4±15.9 p=0.116
Regular exercising			
Yes No	12(14.6) 70(85.4)	57.2±19.9 46.6±18.2 p=0.080	45.7±14.1 36.6±15.3 p=0.023
Opinion on BMT			
I believe it will be remedial Remedial and many side effects I don’t believe it will be remedial	32(39.0) 48(58.5) 2(2.5)	47.0±17.1 49.8±19.6 25.0±0.6 p=0.142	38.0±14.6 38.8±15.6 15.5±5.7 p=0.079
Received training on BMT			
Yes No	39(47.6) 43(52.4)	52.4±19.4 44.2±17.4 p=0.050	41.5±17.1 34.7±13.1 p=0.046
Total	82(100.0)	48.1±18.7	37.9±15.4

n: number, MCS: mental component summary, PCS: physical component summary, SD: Standard deviation

**Table-III:** Comparison of patients’ mean scores of Edmonton Symptom Assessment Scale and Quality of Life before and after stem cell transplantation

*Symptoms * *and quality of life*	*Pre-Transpl. * *Mean±SD *	*Post-Transpl. * *Mean±SD *	*p*
Symptoms			
Pain	2.3±1.7	3.7±1.8	0.000
Fatigue	4.2±2.0	5.6±1.8	0.000
Nausea	2.3±1.7	3.7±1.7	0.000
Sadness	3.6±1.6	4.0±2.1	0.008
Anxiety	3.7±1.8	4.1±1.9	0.104
Insomnia	2.9±1.7	3.9±1.8	0.000
Loss of appetite	2.6±1.7	3.8±1.7	0.000
Feeling well	2.6±2.0	3.8±1.7	0.000
Shortness of breath	0.6±1.2	1.1±1.8	0.000
Changes in skin and nails	3.3±1.2	4.0±1.1	0.000
Stomatitis	1.0±1.4	2.2±1.6	0.000
Numbness in hands	1.1±1.7	1.7±2.0	0.000
Quality of Life			
Physical	48.1±18.7	44.0±16.5	0.006
Mental	37.9±15.4	33.8±13.4	0.002

**SD: **Standard deviation

**Table-IV:** Relationship between patients’ mean scores of Edmonton Symptom Assessment Scale and Quality of Life before stem cell transplantation

			*Quality of Life*	
	*PCS*		*MCS*	
	*r*	*p*	*r*	*p*
Pain	-0.634	0.000	-0.473	0.000
Fatigue	-0.597	0.000	-0.420	0.000
Nausea	-0.460	0.000	-0.362	0.002
Sadness	-0.531	0.000	-0.415	0.000
Anxiety	-0.492	0.000	-0.394	0.000
Insomnia	-0.493	0.000	-0.382	0.000
Loss of appetite	-0.546	0.000	-0.420	0.000
Feeling well	-0.645	0.000	-0.532	0.000
Shortness of breath	-0.539	0.000	-0.456	0.000
Changes in skin and nails	-0.644	0.000	-0.271	0.000
Stomatitis	-0.644	0.000	-0.458	0.000
Numbness in hands	-0.586	0.000	-0.434	0.000

MCS: mental component summary, PCS: physical component summary.

Some 31.7% of the patients included in the study had acute myeloid leukemia, 79.3% of them had their time of diagnosis more than six months ago, 35.4% were in stage III, and 57.3% were administered allotransplantation, 74.4% of the patients thought that their disease required a long-term treatment, 86.6% stated that their body image has changed, 64.6% had worries about the future, 58.5% thought that stem cell transplantation had many remedial and side effects and 52.4% did not receive any training on stem cell transplantation.

Those who were diagnosed with Hodgkin lymphoma and were at stage I had the lowest mean scores of physical quality of life and those with non-Hodgkin lymphoma and were at stage III had the lowest mean scores mental quality of life. Those with more than six months of diagnosis time and those who were administered autologous transplantation had lower mean scores of both physical and mental quality of life. Those who said that the disease required long-term treatment, those who stated that they had a change in their body image, those who had worries about the future, and those who did not believe that stem cell transplantation would be remedial had lower mean scores of both physical and mental quality of life (Table-II).

The patients had increased mean scores of pain, fatigue, nausea, depression, anxiety, drowsiness, loss of appetite, decreased sense of well-being and shortness of breath, skin and nail changes, stomatitis, and numbness in the hands and decreased mean scores of physical and mental quality of life after the stem cell transplantation as compared to before it. The changes in the symptoms other than anxiety and in quality of life were found to be highly significant (p<0.05) (Table-III).

A negative correlation was found between the symptoms experienced by the patients and their quality of life; as the symptoms increased, their quality of life declined (p<0.05) (Table-IV).

## DISCUSSION

In this study, the symptoms experienced by the patients with hematological problems, their quality of life and the relationship between these symptoms and quality of life before and after stem cell transplantation were evaluated. The study was performed a month before the stem cell transplantation and a month after it. This was because there were reports from other studies that patients had intense problems in these periods and such problems affected patients adversely in many respects.^10^^,^^11^

As a result of the study, the patients were observed to have quite low mean scores of physical and mental quality of life a month before transplantation and this was more apparent in their mental quality of life scores. The symptoms most intensely experienced by the patients were fatigue, anxiety, sadness, skin and nail changes, drowsiness, loss of appetite, decreased sense of well-being, pain, nausea, numbness in the hands, stomatitis, and shortness of breath in that order. Looking at the results a month after transplantation, the mean scores of both physical and mental quality of life further declined as compared to before transplantation and the severity of all the symptoms starting from fatigue increased. We think that the increase in the severity of symptoms was the cause of decreased quality of life of the patients after transplantation. In fact, it was also observed in other studies performed at various time intervals after transplantation that patients generally had low quality of life^12^^-^^14^, they had the lowest quality of life particularly in the second week of transplantation^10^ and complications and some health-related problems were experienced in the early stage (first 100 days), mid stage (100 days-1 year) and in the long-term (later than a year).^11^ Again in this period, patients were reported to have experienced changes in especially their physical symptoms, body images and sexual lives^15^^-^^17^, further worsening of their health conditions, job losses^17^, social problems^13^ and financial problems.^18^ In the post-transplantation period, patients commonly had fatigue^11^^,^^14^^,^^15^ and faced problems such as anxiety, depressive symptoms^17^, loss of energy, headache, dizziness^19^, concern for the future, and fear of relapse.^13^

Studies have showed that some symptoms such as physical well-being^20^, sleeping problems, pain^21^, and anxiety were among the factors that affected quality of life the most.^1^ The fact that those who experienced more intense symptoms had worse quality of life in our study supports the results of these studies.

Apart from these problems experienced in the course of both the disease and treatment, some characteristics of patients also affect their quality of life. It was observed in our study that those of advanced age, women, those with poor economic status and those with lower level of education had poorer quality of life. Other study also revealed that women and those of advanced age had lower quality of life.^12^ However, there are also studies that have not found any relationship between parameters such as age, gender and education, and quality of life.^13^^,^^17^ Therefore, it is very important for the members of health staff to evaluate socio-demographic and regional characteristics alongside symptom control during treatment and care, and offer guidance in solving possible problems of patients.

As reported it is important that every patient is evaluated individually in terms of all of their functions and quality of life with a multidisciplinary team approach^21^ before and after transplantation.^22^ It was reported that particularly after transplantation it was quite effective when the symptoms and primarily fatigue were alleviated to enable patients continue with their daily life, they are helped to increase their quality of life.^15^ Additionally, if the members of healthcare staff teach patients about coping skills and appropriate nursing interventions are made after an assessment is done with a holistic approach, this will have a positive impact on their quality of life.

## CONCLUSION

It was found that the patients had decreased physical and mental quality of life both before and after transplantation and this decline further increased in the first month after transplantation. Similarly, all the symptoms were experienced more intensely after stem cell transplantation as compared to before it; female gender, advanced age, and low level of education and economic status affected quality of life negatively. As the severity of symptoms increased, quality of life deteriorated. For this reason, nurses have a great responsibility for assessing and managing symptoms in regular intervals to support quality of life especially immediately before and after transplantation without overlooking the socio-demographic variables.

## Authors Contributions

NO, EK, MP: Study design.

EK, NO, OO: Data collection and analysis. 

NO, OO, EK, GK: Manuscript preparation.

## References

[1] Gregurek R, Brajkovic L, Kalenic B, Bras M, Persic-Brida M (2009). Five years study on impact of anxiety on quality of life in patients treated with bone marrow transplantation. Psychiatr Danub.

[2] Yilmaz MC (2004). Peripheral stem cell transplantation practices and nursing care in pediatric patients. XIII. TPOG National Pediatric Cancer Congress. Nursing Program.

[3] Kapucu SS, Karaca Y (2008). Patient assessment and care in stem cell transplantation. CU Nursing J.

[4] Barrett SC, Huffman FG (2011). Comparison of self-perceived weight and desired weight versus actual body mass index among adolescents in Jamaica. Rev Panam Salud Publica.

[5] Bruera E, Kuehn N, Miller MJ, Selmser P, Macmillan K (1991). Edmonton symptom assessment system (ESAS): a simple method for the assessment of palliative care patients. Palliative Care.

[6] Sadirli SK, Unsar S (2009). Edmonton Symptom Assessment Scale for Patients with Cancer: Its Validity and Reliability in Turkish. Firat Health Services J.

[7] Kurt S, Unsar S (2011). Assessment of symptom control in patients with cancer in Northwestern Turkey. European J Oncol Nursing.

[8] Ware JE, Sherbourne DC (1992). The MOS 36 item short-form health survey (SF-36). Medical Care.

[9] Pinar R (1995). A new aspect of health-related research: quality of life, evaluation of reliability and validity of a quality of life survey in patients with chronic diseases. Nursing Bulletin.

[10] Frodin U, Borjeson S, Lyth J, Lotfi K (2011). A prospective evaluation of patients' health-related quality of life during auto-SCT: a 3-year follow-up. Bone Marrow Transplant.

[11] Bevans MF, Mitchell SA, Marden S (2008). The symptom experience in the first 100 days following allogeneic hematopoieticstem cell transplantation (HSCT). Support Care Cancer.

[12] Chiodi S, Spinelli S, Ravera G, Petti AR, Van Lint MT, Lamparelli T (2000). Quality of life in 244 recipients of allogeneic bone marrow transplantation. Br J Haematol.

[13] Hensel M, Egerer G, Schneeweiss A, Goldschmidt H, Ho AD (2002). Quality of life and rehabilitation in social and professional life after autologous stem cell transplantation. Ann Oncol.

[14] Grulke N, Albani C, Bailer H (2012). Quality of life in patients before and after haematopoietic stem cell transplantation measured with the European Organization for Research and Treatment of Cancer(EORTC) Quality of Life Core Questionnaire QLQ-C30. Bone Marrow Transplant.

[15] Whedon M, Stearns D, Mills LE (1995). Quality of life of long-term adult survivors of autologous bone marrow transplantation. Oncol Nurs Forum.

[16] Sanders JE, Hoffmeister PA, Storer BE, Appelbaum FR, Storb RF, Syrjala KL (2010). The quality of life of adult survivors of childhood hematopoietic cell transplant. Bone Marrow Transplant.

[17] Mosher CE, DuHamel KN, Rini C, Corner G, Lam J, Redd WH (2011). Quality of life concerns and depression among hematopoietic stem cell transplant survivors. Support Care Cancer.

[18] Kopp M, Holzner B, Meraner V, Sperner-Unterweger B, Kemler G, Nguyen-Van-Tam NP (2005). Quality of life in adult hematopoietic cell transplant patients at least 5 years after treatment: a comparison with healthy controls. Eur J Haematol.

[19] Van Agthoven M, Vellenga E, Fibbe WE, Kingma T, Uyl-de Groot CA (2001). Cost analysis and quality of life assessment comparing patients undergoing autologous peripheral blood stem cell transplantation or autologous bone marrow transplantation for refractory or relapsed non-Hodgkin's lymphoma or Hodgkin's disease. Eur J Cancer.

[20] Heinonen H, Volin L, Uutela A, Zevon M, Barrick C, Ruutu T (2001). Quality of life and factors related to perceived satisfaction with quality of life after allogeneic bone marrow transplantation. Ann Hematol.

[21] Kav S, Aslan O, Tekin F, Yesil H, Meral C, Ozturk U (2009). Quality of life and difficulties of patients encountered after autologous stem cell transplantation. J Buon.

[22] Saleh US, Brockopp DY (2001). Quality of life one year following bone marrow transplantation: psychometric evaluation of the quality of life in bone marrow transplant survivors tool. Oncol Nurs Forum.

